# Neutrophils induce macrophage anti-inflammatory reprogramming by suppressing NF-κB activation

**DOI:** 10.1038/s41419-018-0710-y

**Published:** 2018-06-04

**Authors:** John A. Marwick, Ross Mills, Oliver Kay, Kyriakos Michail, Jillian Stephen, Adriano G. Rossi, Ian Dransfield, Nikhil Hirani

**Affiliations:** 1 The MRC Centre for Inflammation Research, Queen’s Medical Research Institute, University of Edinburgh, 47 Little France Crescent, Edinburgh, EH16 4TJ UK; 2 Advanced Therapeutics, Cellular Therapies, Jack Copeland Centre, Scottish National Blood Transfusion Service, Edinburgh, EH14 4BE UK

## Abstract

Apoptotic cells modulate the function of macrophages to control and resolve inflammation. Here, we show that neutrophils induce a rapid and sustained suppression of NF-κB signalling in the macrophage through a unique regulatory relationship which is independent of apoptosis. The reduction of macrophage NF-κB activation occurs through a blockade in transforming growth factor β-activated kinase 1 (TAK1) and IKKβ activation. As a consequence, NF-κB (p65) phosphorylation is reduced, its translocation to the nucleus is inhibited and NF-κB-mediated inflammatory cytokine transcription is suppressed. Gene Set Enrichment Analysis reveals that this suppression of NF-κB activation is not restricted to post-translational modifications of the canonical NF-κB pathway, but is also imprinted at the transcriptional level. Thus neutrophils exert a sustained anti-inflammatory phenotypic reprogramming of the macrophage, which is reflected by the sustained reduction in the release of pro- but not anti- inflammatory cytokines from the macrophage. Together, our findings identify a novel apoptosis-independent mechanism by which neutrophils regulate the mediator profile and reprogramming of monocytes/macrophages, representing an important nodal point for inflammatory control.

Neutrophils were once thought to be relatively monofunctional cells, with a role comprising of an early recruitment to the site of injury to kill and remove infectious agents. However, the diversity of their immunomodulatory functions has become increasingly apparent^1^, particularly through apoptosis which provides a powerful anti-inflammatory signal^2^. The most intensely studied anti-inflammatory action of apoptotic neutrophils is the interaction with macrophages, in which the macrophage mediator profile switches from pro- to anti-inflammatory^3,4^. The resultant suppression of inflammatory cytokines such as TNF and CXCL-8^4^ and increased negative/positive feedback loops from anti-inflammatory mediators, including TGFβ^5^, IL-10^6^ and Resolvins^7^ are pivotal in driving an anti-inflammatory/pro-resolving microenvironment that favours tissue repair^8^. More recent studies have shown that viable neutrophils also modulate the inflammatory actions of surrounding cells through the release of microvesicles^9^. The potential for neutrophils to exert control of macrophage inflammatory functions independently of apoptosis suggests a unique relationship where, far from being functionally limited, neutrophils form an integral part of the mechanism for control of inflammation. However, our understanding of the intracellular signalling that neutrophils elicit in macrophages remains unclear and is confounded by a lack of a direct comparison between the immunomodulatory effects mediated by apoptotic and viable neutrophils.

NF-κB is a key regulator of inflammatory signalling^10^ and an important molecular determinant of macrophage phenotype through induction of inflammatory mediators including TNF, IL-6, CXCL-8, IL-1β, IL-12 and COX^11^. Studies focusing on the effect of apoptotic neutrophils show a reduction in TLR4-induced release of inflammatory cytokines from macrophages (e.g., TNF and CXCL-8)^4^. This implicates an involvement of NF-κB, which is activated through canonical TLR4 signalling to facilitate the transcription of inflammatory mediators such as TNF and CXCL-8^10,11^. However, a direct involvement for NF-κB has not been demonstrated. Studies using murine macrophage cell lines co-cultured with apoptotic cell targets have suggested that although DNA binding of NF-κB is unaffected^12^, the transcriptional activity of NF-κB sites is reduced indirectly through interference with a common transcriptional co-activator such as CBP or p300^13^. However, there are no studies relating to whether viable and apoptotic neutrophils exert distinct effects on NF-κB activation to modulate macrophage mediator profiles and whether these regulatory mechanisms are present in human cells. In this study, we use primary human cells to address the key questions of whether neutrophils modulate the inflammatory signalling of macrophages by suppressing NF-κB activation and whether this modulation differs once the apoptotic program is engaged.

## Results

### Apoptotic and viable neutrophils induce a near identical change in the cytokine profile released by macrophages

**Fig. 1:**
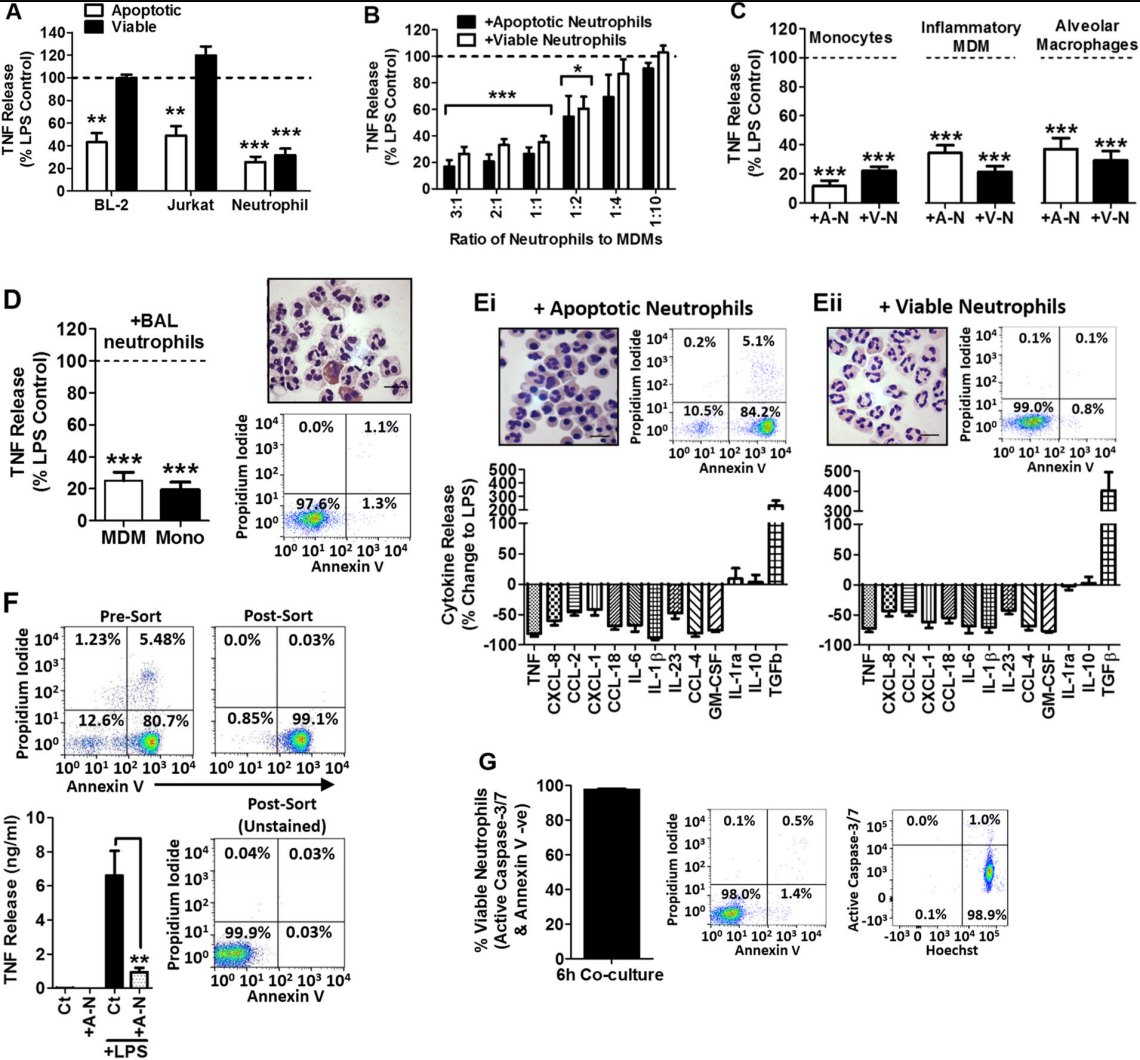
Neutrophil-mediated changes to the macrophage inflammatory cytokine profile. **a** LPS-induced (1 ng/ml; 6 h) TNF release from MDM co-cultured with viable and apoptotic BL-2 cells, Jurkat cells (both *n* = 6) and neutrophils (*n* = 10). **b** LPS-induced (1 ng/ml; 6 h) TNF release from co-cultures of MDM with apoptotic (closed bars) and viable (open bars) neutrophils at the ratio of neutrophil to MDM as indicated (*n* = 4). **c** LPS-induced (1 ng/ml; 6 h) TNF release from monocytes (*n* = 12), INFγ (20 ng/ml) primed MDM (*n* = 6) and alveolar macrophages (*n* = 11) co-cultured with apoptotic or viable neutrophils. **d** LPS-induced (1 ng/ml; 6 h) TNF release from MDM and monocytes co-cultured with neutrophils isolated from BAL (*n* = 4): cytocentrifugation preparation of BAL neutrophils stained with Diff-Quik shows the multi-lobed morphology of viable neutrophils, cells stained pink are eosinophils. Flow cytometry of BAL neutrophils stained with annexin V and propidium iodide where the upper left quadrant represents cell debris, the upper right quadrant represents necrotic cells, the lower right quadrant represents apoptotic cells and the lower left represents viable cells. **e** LPS-induced (1 ng/ml; 6 h) cytokine profiles from MDM co-cultured with apoptotic (i) and viable (ii) neutrophils (*n* = 6-12). **f** Basal and LPS-induced (1 ng/ml; 6 h) TNF release from MDM co-cultured with apoptotic neutrophils purified by cell sorting annexin V^+^/propidium iodide^-^ cells (*n* = 7). Neutrophils are negative for bound annexin V after sorting due to reduction of the extracellular Ca^2+^ concentration and thus annexin V does not affect the subsequent incubation with MDM. **g** Percentage of viable neutrophils that become apoptotic, as measured by either (i) annexin V^+^ or (ii) activation of caspase 3/7, over the period of a 6 h co-culture with MDM and LPS (1 ng/ml; *n* = 6). All n-numbers represent data derived from separate healthy donors with data plotted as mean ± s.e.m. Neutrophil to MDM or monocyte ratio in co-cultures were 3:1, unless otherwise stated. Image scale bars = 10 μm. ***P > 0.001, **P > 0.01, *P > 0.05 using paired *t*-test (**a**–**d**) or ANOVA with Tukey post-hoc test (**f**)*.* Ct Control, MDM monocyte derived macrophage, A-N apoptotic neutrophil, V-N viable neutrophil, Mono monocyte

Firstly, we sought to address the question of whether the LPS-induced macrophage cytokine profile was modulated by co-culture with either viable or apoptotic neutrophils and, if so, whether they exert differential effects? We measured the release of TNF induced by LPS from human monocyte-derived macrophages (MDMs) which were co-cultured for 6 h with either viable or apoptotic populations from three distinct human immune cell lineages; blood neutrophils, T cells (Jurkat cell line) and B cells (BL-2 cell line). Co-culture of MDM with apoptotic cells significantly reduced the release of TNF from the MDMs, regardless of cell lineage (Fig. 1a). In contrast, only viable neutrophils significantly reduced the release of TNF when co-cultured with MDM (Fig. 1a). Importantly, the potency of the suppression induced by viable neutrophils was similar to that induced by apoptotic neutrophils (Fig. 1b). We extended our analysis to include blood monocytes, inflammatory (IFN-γ) primed MDMs and primary human alveolar macrophages (Fig. 1c), all of which showed a reduction in LPS-induced TNF release when co-cultured in the presence of either viable or apoptotic neutrophils. Thus, the suppression of TNF release induced by viable and apoptotic neutrophils was not restricted to a particular monocyte/macrophage differentiation/activation status. To exclude the possibility that these observations reflected a unique property of blood neutrophils, we next co-cultured monocytes and MDMs with viable neutrophils isolated from the bronchoalveolar lavage (BAL) of patients with lung inflammation which represent a true activated neutrophil from a site of inflammation. In these experiments, the lung neutrophils (>97% viable) were found to induce the same suppressive effects on monocyte and macrophage TNF release (Fig. 1d), confirming the potential for viable neutrophils to modulate cytokine production at an inflammatory site and the validity of using blood neutrophils.

Comparison of the spectrum of changes to the MDM cytokine profile following co-culture with apoptotic (Fig. 1e–i) or viable neutrophils (Fig. 1e–ii), revealed a common pattern; the release of inflammatory cytokines such as TNF, CXCL-8 and CCL-2 were suppressed, whilst the release of anti-inflammatory cytokines such as IL-10, IL-1ra and TGFβ were either unchanged or elevated. The near identical alterations to MDM cytokine profile induced by viable and apoptotic neutrophils raised the question of whether the presence of residual viable cells in the apoptotic neutrophil preparations could account for these results. Highly purified apoptotic neutrophil populations (isolated by cell sorting and containing <1% viable neutrophils; Fig. 1f) were highly effective at reducing the release of TNF from LPS-stimulated MDMs, definitively demonstrating that apoptotic neutrophils alone were capable of modulating macrophage cytokine production (Fig. 1f). Conversely, we found that <2% of the neutrophil population underwent apoptosis, as assessed by caspase 3/7 activation and staining for annexin V and propidium iodide (Fig. 1g) during the 6 h co-culture period. At the neutrophil to MDM ratio of 3:1 used in these experiments, the effective ratio of apoptotic neutrophils to MDMs would be equivalent to 1:16.7 which we have shown is too low to suppress MDM cytokine production (Fig. 1b). These data definitively demonstrate that the modulation of the LPS-induced cytokine profile of monocyte/macrophage by neutrophils is independent of apoptosis.

### Neutrophils suppress inflammatory macrophage cytokine production in response to TLR4, TNFR1 and IL-1R ligands

**Fig. 2:**
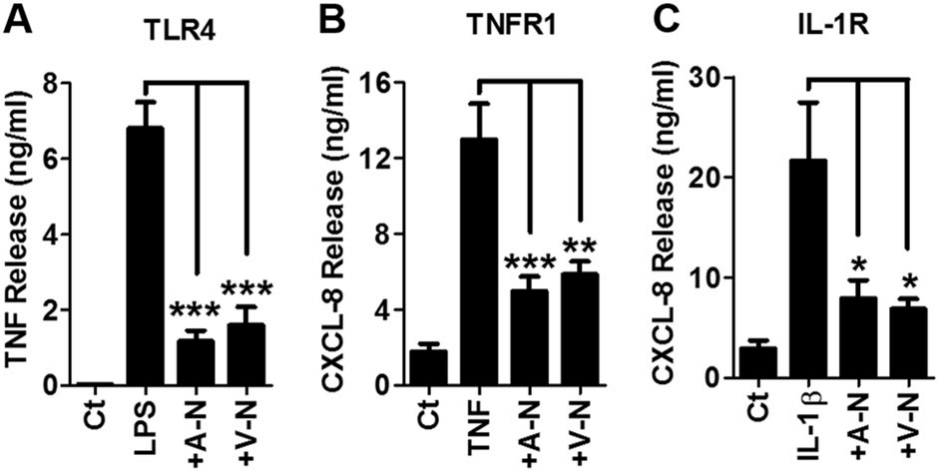
Neutrophil-mediated suppression of macrophage inflammatory cytokine release in response to different inflammatory signalling. The release of TNF (**a**), CXCL-8 (**b**, **c**) from MDM co-cultured with neutrophils and stimulated with LPS (1 ng/ml), TNF (20 ng/ml) and IL-1β (10 ng/ml), respectively, for 6 h. Data derived from 8 separate healthy donors and plotted as mean ± s.e.m. ***P > 0.001, **P > 0.01, *P > 0.05 using ANOVA with Tukey post-hoc test. Ct control, A-N apoptotic neutrophil, V-N viable neutrophil

Our demonstration that both apoptotic and viable neutrophils induce a common suppression of LPS-induced cytokine release from MDM raised the question as to whether this effect was specific to TLR4-mediated signalling or extended to other inflammatory signalling pathways. The issue of specificity is physiologically important, as suppression of a broad spectrum of inflammatory signalling pathways would exert effective control of macrophage cytokine production at an inflammatory site. We therefore tested the effects of neutrophil co-culture on two distinct receptor pathways in macrophages; IL-1R and TNFR1. MDM release of CXCL-8 following treatment with either IL-1β or TNF was suppressed following co-culture with apoptotic or viable neutrophils (Fig. 2b, c). These data demonstrate that apoptotic and viable neutrophils regulate macrophage cytokine production through a common mechanism to a range of pro-inflammatory agonists.

### The neutrophil-mediated repression of inflammatory cytokines in MDM is linked to a rapid reduction of NF-κB activation

**Fig. 3:**
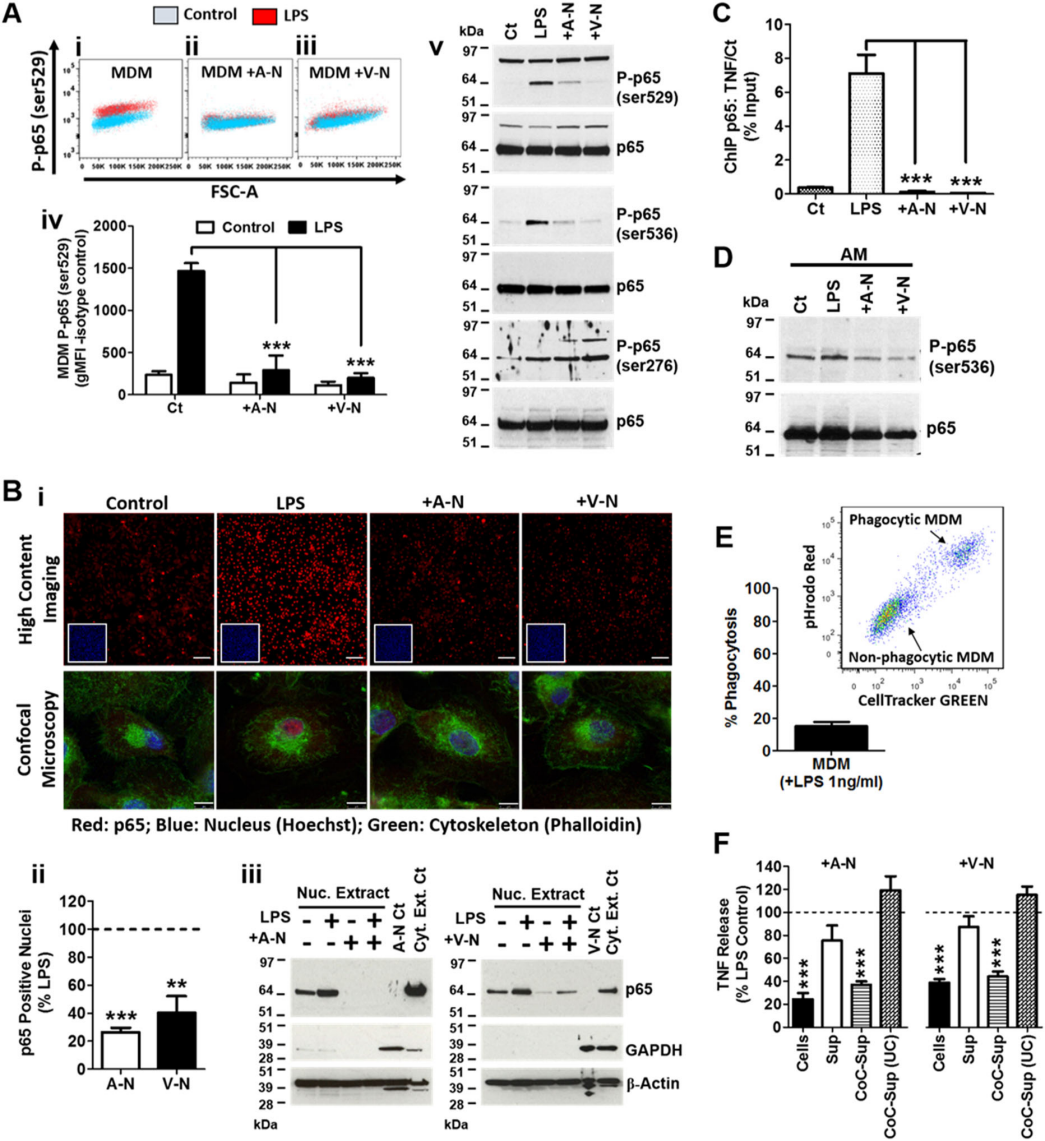
The impact of neutrophils on NF-κB activity in MDM. **a** LPS-induced (1 ng/ml; 20 min) p65 phosphorylation measured by (i-iv) flow cytometry (serine 529; *n* = 4) and (v) immunoblotting (serine 536, 529 and 276; *n* = 3) in MDM co-cultured with apoptotic or viable neutrophils (total time point: 30 min). **b** LPS-induced (1 ng/ml; 20 min) p65 nuclear translocation assessed by (i) quantitative high-content imaging and confocal microscopy (scale bars 100 μm and 7.5 μm, respectively), (ii) quantification of p65 nuclear translocation from high-content imaging (*n* = 8) and (iii) immunoblotting in MDM co-cultured with apoptotic and viable neutrophils (*n* = 3). Full images of individual stains and merged staining panels are available in supplemental data (supplemental data Fig. 1). **c** LPS-induced (1 ng/ml; 1 h) p65 binding to the TNF promotor in MDM co-cultured with apoptotic or viable neutrophils (*n* = 3). **d** LPS-induced (1 ng/ml; 20 min) p65 phosphorylation in alveolar macrophages (*n* = 3). **e** Phagocytosis of apoptotic neutrophils labelled with CellTracker Green and pHrodo Red by MDM at 40 min of co-culture in the presence of LPS (1 ng/ml; *n* = 14). **f** LPS-induced (1 ng/ml; 6 h) TNF release from MDM co-incubated with either apoptotic or viable neutrophils (cells), the supernatants from apoptotic or viable neutrophils cultured alone for 6 h (Sup) or the supernatants from apoptotic or viable neutrophils co-cultured with MDM for 6 h before (CoC-Sup) or after (CoC-Sup (UC) ultracentrifugation at 100,000 g for 1 h (*n* = 4). All n-numbers represent data derived from separate healthy donors with data plotted as mean ± s.e.m. ***P > 0.001, *P > 0.05 using paired *t*-test*.* A-N apoptotic neutrophil, V-N viable neutrophil, ser serine, Ct control, Nuc nuclear, Cyt. Ext. Ct cytoplasmic extract control, AM alveolar macrophage

NF-κB is involved in the downstream signalling via TLR-4, IL-1R and TNFR1 and subsequent transcriptional regulation of the inflammatory cytokines that were suppressed following co-culture of macrophages with neutrophils. We therefore investigated the impact of apoptotic and viable neutrophils on the activation of NF-κB activation in MDM by assessing the changes in the phosphorylation status of p65. Using a quantitative flow cytometric assay, we could demonstrate that LPS induced an increase in phosphorylated p65 (P-p65) at serine 529 for the entire MDM population (see representative histograms comparing unstimulated (blue) and LPS-stimulated (red) cells; Fig. 3a–i). This effect was rapid, with increased levels of P-p65 seen within 30 min. However, for MDM incubated with either apoptotic (Fig. 3a–ii) or viable neutrophils (Fig. 3a–iii), no increase in P-p65 fluorescence was observed following LPS treatment. Quantification of the geometric mean fluorescence for unstimulated and LPS-stimulated MDM populations revealed that LPS induced a ~6-fold increase in the levels of P-p65 which was inhibited by ~90% in the presence of either apoptotic or viable neutrophils (Fig. 3a–iv). Moreover, these data clearly demonstrate that co-incubation with neutrophils acted to rapidly (<30 min) suppress NF-κB activation across the entire population rather than any particular subset of MDM. Immunoblot analysis confirmed the reduction of P-p65 at serine 529 and show that P-p65 at serine 536 was also reduced following neutrophil co-culture, whilst P-p65 at serine 276 was unaffected (Fig. 3a–v). Phosphorylation of p65 at serines 529 and 536 is critical for nuclear translocation of NF-κB^14–16^. Consistent with this, LPS-driven nuclear translocation of p65 in MDMs was abolished in the presence of either apoptotic or viable neutrophils as determined by confocal microscopy (Fig. 3b–i), quantitative high content imaging (Fig. 3b–i-ii) and immunoblot analysis of nuclear extracts (Fig. 3b–iii). These data clearly show that the activation of NF-κB in MDMs was reduced by the presence of either apoptotic or viable neutrophils. We then assessed the level of p65 bound to the TNF promoter by chromatin immunoprecipitation (ChIP) to determine the relationship between NF-κB activity and the reduction in the release of inflammatory cytokines (Fig. 3c). This analysis demonstrated that the level of p65 bound at the TNF promoter induced by LPS was significantly reduced by the presence of either apoptotic or viable neutrophils (Fig. 3c). Consistent with the MDM model, this neutrophil-induced suppression of NF-κB activation was also seen in primary human alveolar macrophages (AM; Fig. 3d).

Phagocytosis of apoptotic cells is believed to be an important step in macrophage reprogramming. However, viable neutrophils are not phagocytosed and MDM co-incubated with apoptotic neutrophils labelled with CellTracker Green and the pH sensitive pHrodo RED (to indicate phagolysosme pH) show less than 20% phagocytosis by 40 min (Fig. 3e), markedly contrasting the abolition of NF-κB phosphorylation in ~90% MDM following 30 min of co-culture (Fig. 3a). To establish if contact was required for neutrophils to supress the inflammatory mediator production from macrophages, or if released mediators were responsible, macrophages were treated with supernatant from viable and apoptotic-MDM co-culture. The supernatant suppressed the release of TNF from MDM to an extent that was comparable with co-culture. The suppression of TNF release by supernatants was abolished by ultracentrifugation (Fig. 3f), indicating that larger extracellular vesicles and/or mediator complexes were responsible rather than soluble mediators such as TGFβ which has previously been proposed to play a role^5^. Indeed, the levels TGFβ were unchanged by ultracentrifugation and the addition of exogenous TGFβ had no significant impact on LPS-induced release of TNF from MDMs (supplementary data fig. 4). These data, along with several previous studies^17,18^, suggest that TGFβ alone does not induce this suppression. Importantly, supernatant from apoptotic or viable neutrophils alone had no significant effect (Fig. 3f). Together, these data suggest that neither phagocytosis nor soluble mediators account for the inhibition of NF-κB or the subsequent release of TNF induced by either apoptotic or viable neutrophils. Instead, it is likely that the release of extracellular vesicles and/or mediator complexes upon co-culture of MDM and neutrophils is required. These data identify a direct link between a rapid suppression of NF-κB activation and the reduction in LPS-induced release of inflammatory cytokines from MDM by apoptotic and viable neutrophils which is independent of phagocytosis.

### The neutrophil-induced suppression of macrophage NF-κB activation and inflammatory cytokine release is sustained

**Fig. 4:**
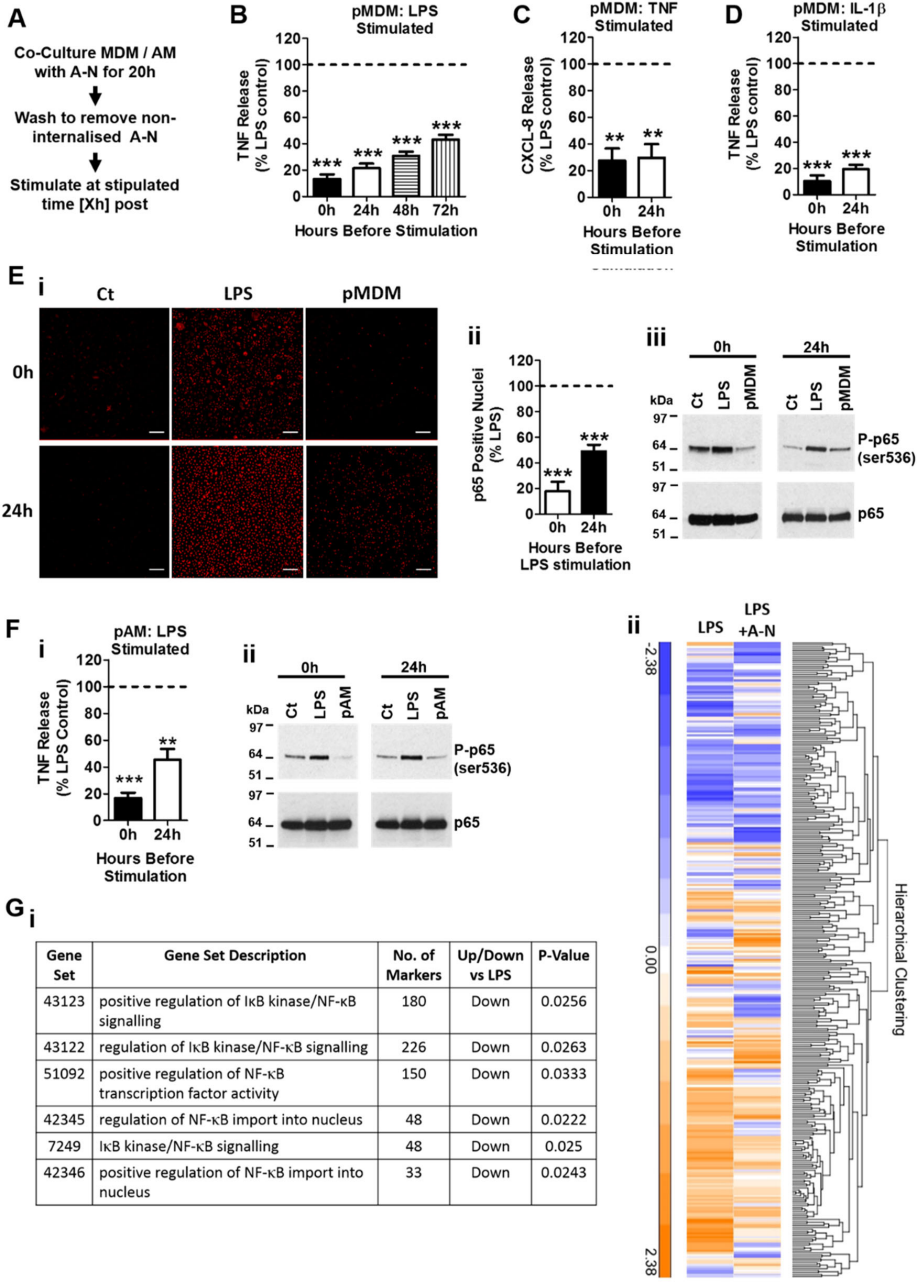
Sustained influence of apoptotic neutrophils on the macrophage cytokine profile. **a** Work flow for MDM and AM culture and stimulation post co-culture (pMDM and pAM, respectively). **b** LPS-induced (1 ng/ml, 6 h) TNF release from pMDM at 0, 24, 48 and 72 h (*n* = 6). **c**, **d** Release of CXCL-8 (**c**) and TNF (**d**) from pMDM stimulated with TNF (20 ng/ml) and IL-1β (10 ng/ml), respectively, for 6 h at 0 or 24 h (*n* = 4). **e** high-content imaging (i; scale bar 100 μm) and quantification (ii, *n* = 6) of p65 translocation in pMDM stimulated with LPS (1 ng/ml, 1 h) at 0 h and 24 h, (iii) LPS-induced (1 ng/ml, 30 min) p65 phosphorylation (serine 536) in pMDM at 0 h or 24 h (*n* = 3). Full images of individual stains and merged staining panels from high content imaging are available in supplemental data (supplemental data Fig. 2). **f** TNF release (i) and p65 phosphorylation (serine 536) (ii) from pAM at 0 and 24 h (*n* = 11 and 3, respectively). **g** Table (i) of significantly different NF-κB-related pathways (with a frequency distribution ratio of less than 0.2) identified by gene set enrichment analysis (GSEA) of an affymetrix 2.1 ST array and (ii) Hierarchical clustering of the combined genes (326) from these significant NF-κB pathways from LPS-stimulated MDM (1 ng/ml; 9 h) cultured with or without apoptotic neutrophils (*n* = 4). All n-numbers represent data derived from separate healthy donors with data plotted as mean ± s.e.m. ***P > 0.001, **P > 0.01 using paired *t*-test. A-N apoptotic neutrophil, pMDM MDM post co-culture with apoptotic neutrophils, pAM AM post co-culture with apoptotic neutrophils, Ct control, h hour

Our data demonstrate that viable neutrophils have the potential to modulate monocyte and macrophage inflammatory functions during the early phases of inflammation. However, during the later stages of inflammation, the interaction between macrophages and apoptotic cells will predominate, representing a pivotal step in reprogramming macrophages towards an anti-inflammatory phenotype^8^. To address the key question of whether the modulation of macrophage cytokine profile represents a transient or sustained alteration in macrophage behaviour, MDM were co-cultured with apoptotic neutrophils for 20 h prior to the removal of non-internalised cells (Fig. 4a). The release of inflammatory cytokines was then measured at different time points over a period of 72 h. Our data show that suppression of TNF release in response to TLR4 signalling was maintained for at least 72 h (Fig. 4b). Similarly, suppression of both IL-1R and TNFR1-induced cytokines was also sustained (Fig 4c, d), which effect was mirrored by prolonged suppression of p65 phosphorylation (Fig. 4e). Furthermore, sustained suppression of NF-κB activation and TNF release by apoptotic neutrophils was also observed in AM (Fig. 4f), demonstrating that the MDM model was reflective of primary human macrophages.

We next determined whether a footprint of the apoptotic neutrophil-induced alteration in NF-κB signalling occurred at the transcriptional level. Gene set enrichment analysis (GSEA) of Affymetrix Human Gene 2.1 ST array demonstrated that MDM co-cultured with apoptotic neutrophils have a significant reduction in the gene sets associated with the activation, nuclear translocation and transcriptional activity of NF-κB (Fig. 4g–i). Hierarchical clustering of all the genes contained within these NF-κB pathway gene sets outlines the differences in gene expression induced by LPS in MDM co-cultured with or without apoptotic neutrophils (Fig. 4g–ii). Together, these data suggest that attenuated activation of NF-κB signalling is responsible for both the rapid and sustained suppression of inflammatory cytokine release induced by neutrophils and that this occurs at both at the transcriptional and post-transcriptional level which may be an important contributor to the anti-inflammatory reprogramming of macrophages.

### Neutrophil-induced suppression of TAK1-IKKβ attenuates NF-κB activation

**Fig. 5:**
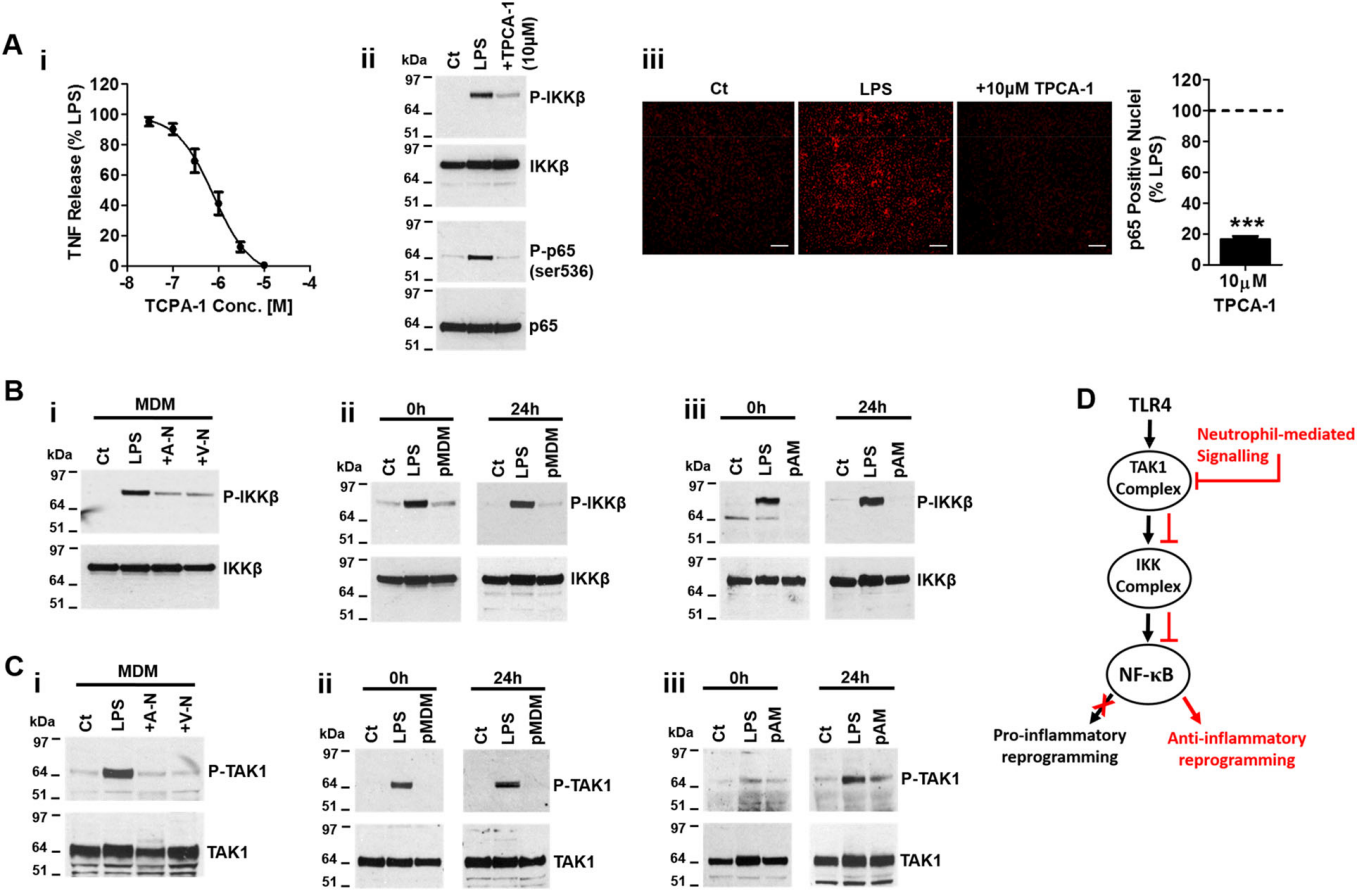
Neutrophil-mediated regulation of TAK1 and IKKβ activation in macrophages. **a** Inhibition of LPS-induced (1 ng/ml) TNF release (*n* = 6) (i), IKKβ and p65 (serine 536) phosphorylation (*n* = 3) (ii) and p65 translocation (*n* = 7; scale bar 100 μm) (iii) in MDM using the IKKβ selective inhibitor TPCA-1. Full images of individual stains and merged staining panels from high content imaging are available in supplemental data. **b** LPS (1 ng/ml; 20 min) induced IKKβ phosphorylation in MDM co-cultured with apoptotic and live neutrophils (i) and in pMDM (ii) and pAM (iii) at 0 h or 24 h (*n* = 3). **c** LPS (1 ng/ml; 20 min) induced TAK1 phosphorylation in MDM co-cultured with apoptotic and live neutrophils (i) and in pMDM (ii) and pAM (iii) at 0 h or 24 h (*n* = 3). **d** Summary flow diagram of proposed inhibition of NF-κB activity in macrophages by neutrophils. All n-numbers represent data derived from separate healthy donors with data plotted as mean ± s.e.m. ***P > 0.001 using paired *t*-test. Conc. Concentration, A-N apoptotic neutrophil, V-N viable neutrophil, Ct Control, h hour

The phosphorylation of p65 at serine 536 is critical for the activation of NF-κB which is in turn facilitated by several upstream kinases, most prominent of which is IKKβ^14,19^. Inhibition of IKKβ using the selective IKKβ inhibitor TPCA-1^20^ suppressed the release of TNF (Fig. 5a–i), reduced P-p65 at serine 536 (Fig. 5a–ii) and inhibited the nuclear translocation of p65 (Fig. 5a–iii) in response to LPS. The manner in which p65 is suppressed in macrophages by direct inhibition of IKKβ is similar to that seen upon co-culture with neutrophils. We therefore examined the impact of neutrophil co-culture on the activation of IKKβ in macrophages. Data shown in Fig. 5 demonstrate that both viable and apoptotic neutrophils potently block the phosphorylation of IKKβ in MDM (Fig. 5b–i) and, importantly, that blockade of IKKβ activation is sustained in both MDM (Fig. 5b–ii) and in AM (Fig. 5b–iii) for at least 24 h, paralleling the sustained suppression of NF-κB (Fig. 4e-g). Thus, the inhibition of IKKβ likely represents a pivotal effector in the neutrophil-induced rapid and sustained blockade of NF-κB activation and inflammatory cytokine release from macrophages.

Phosphorylation of IKKβ is directly mediated by the transforming growth factor beta-activated kinase 1 (TAK1), which links the TLR4, IL-1R and TNFR1 receptor-associated complexes with IKKβ and its downstream effectors^21^. Our data show that both apoptotic and viable neutrophils induce a rapid reduction in the phosphorylation of TAK1 in MDM during co-culture (Fig. 5c–i). The neutrophil-induced blockade in the phosphorylation of TAK1 was sustained in both MDM and AM (Fig. 5c–ii-iii), consistent with the observed sustained suppression of IKKβ (Fig. 5b–ii-iii) and NF-κB (Fig. 4e-g). Together, these data show that neutrophils induce a rapid and sustained inhibition of the TAK1-IKKβ axis (Fig. 5d), representing a central mechanism by which neutrophils facilitate anti-inflammatory reprogramming in macrophages.

## Discussion

**Fig. 6:**
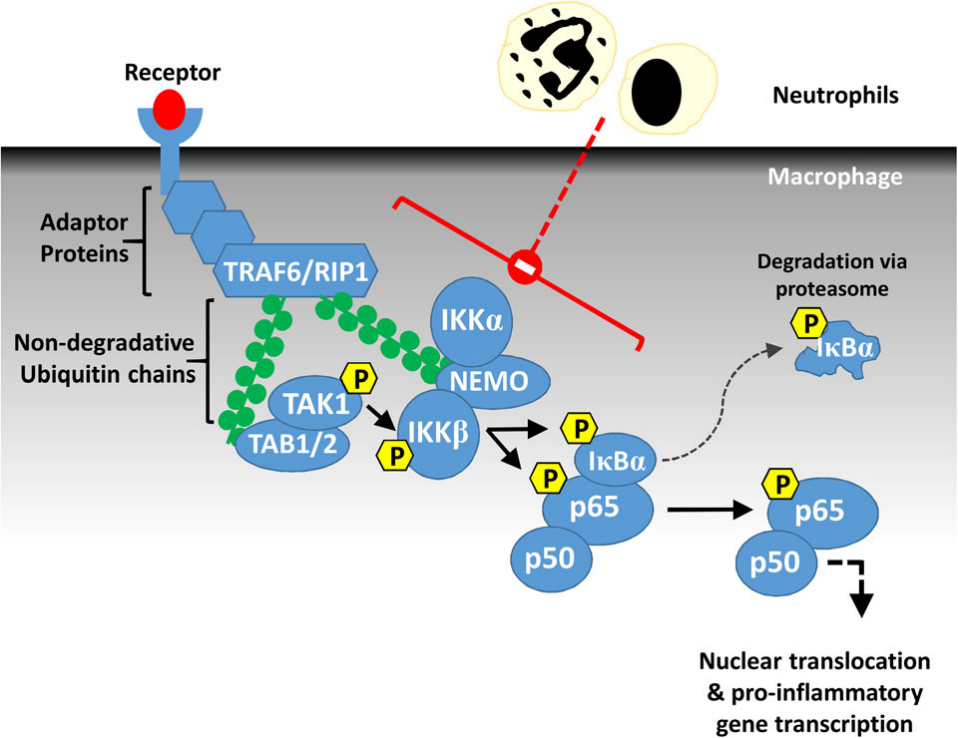
Canonical activation of NF-κB through the TAK1-IKKβ-NF-κB axis. Adaptor proteins are recruited to the cell surface receptors upon its activation. These include the E3 ubiquitin ligase TRAF6 (in the case of TRL4 and IL-1R) and TRAF2/RIP1 (in the case of TNFR1) resulting the formation of non-degradative ubiquitin chains on which TAK1/TAB and IKK complexes are recruited. TAK1 phosphorylates IKKβ which then induces IκB degradation and phosphorylates p65 at serine 536 resulting in NF-κB transactivation and inflammatory gene transcription

Our study shows that both apoptotic and viable neutrophils signal through a common molecular pathway to influence the cytokine profile of macrophages. Central to this mechanism is a direct suppression of NF-κB activation in the macrophage. This reduces the production of pro-inflammatory cytokines such as TNF, CXCL-8 and IL-6 which neutrophils are able to affect across monocytes, as well as classically and non-classically activated macrophage phenotypes. Our data indicate that one of the mechanisms by which neutrophils induce this inactivation of macrophage NF-κB is through blockade of the upstream TAK1-IKKβ signalling axis (Fig. 6). TAK1 is the kinase subunit of the TRAF6-regulated IKK activator 2 (TRIKA2) complex^22^, which along with the regulatory TAB1/2 subunits forms a bridge between activated inflammatory receptors such as TLR4/IL-1R (viaTRAF6), TNFR1 (via receptor interacting protein (RIP) 1) and the IKK complex^22,23^. TAK1 directly phosphorylates IKKβ, which in turn activates NF-κB by inducing IκB degradation and directly phosphorylating p65 at serine 536^21^ which resides within the C-terminal transactivational domain (Fig. 6). P65 phosphorylation is central to NF-κB-mediated gene transcription through facilitating subsequent regulatory modifications such as acetylation^24^ nuclear translocation^25^ and binding to the transcriptional co-activator p300^24^. Therefore, inhibition of TAK1-IKKβ activation in macrophages by neutrophils is likely crucial to the observed abolition of p65 phosphorylation at serine 536 and the subsequent reduction in NF-κB-mediated gene transcription of pro-inflammatory cytokines.

**Fig. 7:**
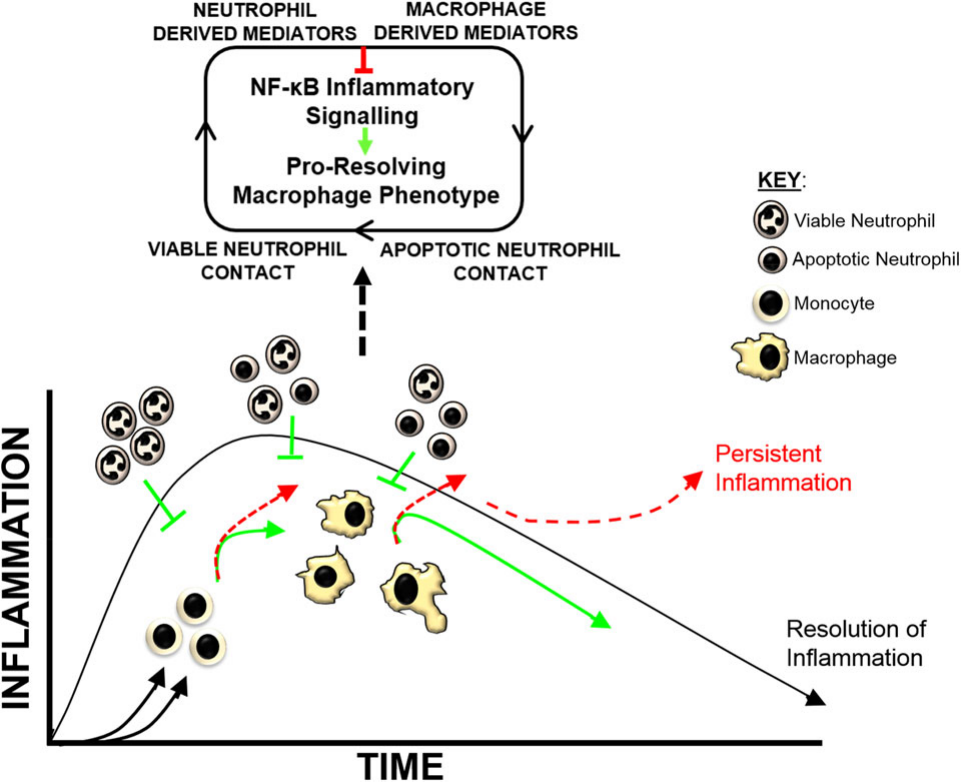
Neutrophil-mediated modulation of monocyte and macrophage mediator profile influences inflammatory control and resolution. Recruitment of neutrophils to the site of injury during an innate inflammatory response has the potential to influence the inflammatory function of recruited monocyte and macrophage populations. These interactions will control the intensity of the inflammatory response. Neutrophils undergoing apoptosis will further regulate responses of monocyte and macrophage populations and drive sustained anti-inflammatory reprogramming, which is pivotal for inflammation resolution and tissue repair

Our studies also demonstrate several important mechanistic insights. First, NF-κB-mediated suppression is induced by either viable or apoptotic neutrophils ensuring that regulatory effects on macrophage inflammatory function occurs independently of changes in neutrophil viability associated with progression of the inflammatory response. This suggests that neutrophils regulate the monocyte and macrophage inflammatory responses from the earliest stages of their recruitment through to the ultimate resolution of inflammation (Fig. 7). Second, we identify a blockade in TAK1-IKKβ-NF-κB activation as a nodal point for neutrophil-mediated control of several inflammatory signalling pathways in macrophages. This provides an important mechanism of regulating macrophage function in either infectious or sterile inflammatory microenvironments where multiple pro-inflammatory signals may be present. Third, we demonstrate that the suppression of TAK1-IKKβ-NF-κB signalling and the consequent inflammatory cytokine release is sustained with a footprint at the transcriptional level is also significant. One implication is that neutrophil-mediated regulation of NF-κB activation in macrophages forms an important part of macrophage anti-inflammatory/pro-resolving reprogramming (Fig. 7). Furthermore, reduction in the phosphorylation of serine 529 on p65 (Fig. 3), indicates that non-canonical pathways such as those that signal through casein kinase II^15^ are also modulated by neutrophils to suppress NF-κB activation in macrophages. The existence of multiple regulatory pathways would be significant; providing a broad repertoire for counter-regulation of the activation of NF-κB in macrophages by neutrophils and again, highlighting the importance of controlling NF-κB activation for anti-inflammatory/pro-resolving reprogramming in macrophages^26^.

Although the mechanism by which neutrophils exert control over the activation of NF-κB and subsequent anti-inflammatory reprogramming in macrophages remains unclear, our observations eliminate some possibilities. First, the rapid suppression of NF-κB in the entire macrophage population rules out a role for post-phagocytosis events. In the time frame covering the suppression of NF-κB, there is no significant internalisation of apoptotic neutrophils by macrophages and even less so by monocytes. Although it is conceivable that the small population of macrophages which have phagocytosed relay a suppressive signal to the surrounding populations, viable neutrophils (which are not internalised) induce equivalent suppression of NF-κB and inflammatory mediator release. We therefore suggest that phagocytosis of apoptotic cells is not required for the observed early suppression of NF-κB, although a role cannot be ruled out for the sustained response. Second, the suppression of TNF release by neutrophil-MDM co-culture supernatants, which is abolished by ultracentrifugation, indicates a requirement for complexes of mediators and/or released extracellular vesicles. Microvesicles released from viable neutrophils exert immunosuppressive activity in vitro^27–29^ and in vivo^9^ when highly concentrated and display several phenotypes^30^. However, our observations suggest that two-way communication between the macrophage and neutrophil is required to induce release of the required mediators. Further detailed studies are needed to define whether cell-cell contact is required for the release of these mediators and the repertoire of receptors/signalling involved. It is also interesting to note that, as with the downstream control of the NF-κB and subsequent alteration in cytokine profile, this requirement of neutrophil-macrophage communication and subsequent vesicle/complex release is independent of apoptosis. Moreover, other mediators including IL-10^6^, TGFβ^5^ and Resolvins^7^ also have a significant impact on macrophage anti-inflammatory reprogramming and are release upon neutrophil-macrophage interactions. Although ultracentrifugation rules out their direct role for the initial suppression of macrophage NF-κB and inflammatory cytokine release these, in combination with released extracellular vesicles, are likely required to orchestrate a spatial and temporal control of the monocyte/macrophage inflammatory phenotype to achieve appropriate reprogramming.

In summary, our data demonstrate a common NF-κB-dependent mechanism by which neutrophils are able to modulate monocyte and macrophage function. Dysregulation of such powerful anti-inflammatory signals are certain to contribute to the pathogenesis of disease via the accumulation of dysfunctional macrophages and the propagation of non-resolving inflammation. Indeed, elevated and prolonged activation of NF-κB in macrophages is seen in a number of chronic inflammatory diseases such as chronic obstructive pulmonary disease^31,32^ and severe asthma^33,34^ and is likely to contribute to the breakdown in inflammatory control. Our study identifies a nodal point in intracellular signalling that could be targeted to bypass the ‘broken’ sections of the signalling and restore the resolution phenotype of dysfunctional macrophages to provide inflammatory control and a pro-resolving microenvironment necessary for inflammation resolution and tissue repair.

## Materials and Methods

### Isolation of neutrophils, monocytes and alveolar macrophages

Peripheral venous blood was taken from healthy human volunteers in accordance with local ethics (AMREC 15-HV-013). Granulocytes and mononuclear cells were isolated from the blood using discontinuous percol gradients as previously described^35^. Granulocytes were 90–95% neutrophils with 5–10% eosinophils as assessed by morphology using cytocentrifuation preparation followed by staining with Diff-Quik (Gentaur Molecular Products) and are referred to throughout the study as neutrophils. Monocytes were purified from the mononuclear cells using a pan monocyte isolation kit (Miltenyi Biotec) and washed 40 min after adhesion to further purify the population from residual lymphocyte and platelet contamination. Alveolar macrophages (AM) and neutrophils were isolated by cell sorting the bronchoalveolar lavage from patient’s undergoing bronchoscopy in accordance with local ethics (AMREC 07/S1102/20; see supplementary data table 1 for patient details).

### Cell culture

Monocytes were cultured in IMDM (Gibco) with 2% autologous human AB serum (AS; Sigma Aldrich) overnight before use. MDM were generated by culturing the monocytes for 5 days in IMDM (2% AS) with 5 ng/ml GM-CSF and 20 ng/ml IL-6 (R&D Systems) to drive differentiation into a macrophage phenotype^36^. During this period, no media was removed, but an additional volume of fresh media (25% of the total volume) containing 2% AS but no cytokines was added on day 3. To induce inflammatory MDM, INFγ (20 ng/ml; R&D Systems) was added 24 h before assay. Immediately before assay the cells are washed with HBSS (Gibco). AM were cultured in IMDM with 2% AU for a minimum of 2 days before treatment. Live neutrophils (from both blood and BAL) were re-suspended in IMDM without serum and used immediately in the appropriate co-culture assay. BL2 Burkitt’s lymphoma cells and Jurkat cells were cultured in RPMI (Gibco) with 10% heat inactivated foetal calf serum (hiFCS; Gibco) and maintained at a density of 0.3–1 million per ml. All cells were cultured using standard culture conditions: 37 degrees with 5% CO_2_.

### Induction of apoptosis

Neutrophils were cultured overnight in IMDM with 1% BSA (Sigma). BL-2 and Jurkat cells were exposed to UV (CL-1000 Ultraviolet Crosslinker; Ultra-Violet Products Ltd) at 300 mJ/cm^2^ and cultured for a further 4 h. Apoptosis was assessed by morphological assessment using cytocentrifugation preparations stained with Diff-Quik and flow cytometry (see below).

### Co-Culture, supernatant and phagocytosis assay

Apoptotic and viable target cell preparations were washed 2 times in HBSS (Gibco) with 2 mM EDTA (Gibco) and then re-suspended in IMDM without serum at the appropriate concentration. Monocyte or MDM cultures were washed in once with HBSS. The apoptotic or viable target cells were added to the monocyte/MDM cultures at a ratio of 3 to 1 for 10 min (unless otherwise stated) in serum free IMDM, followed by LPS stimulation (Escherichia coli O127:B8; Sigma) for the time stated. Where co-culture was for more than 6 h, hiFCS was added to 0.5% at 8 h and maintained for the duration of the assay. For stimulation with TNF or IL-1β (R&D Systems) during co-culture, 0.5% hiFCS was added with TNF/IL-1β.

Co-culture supernatants were generated by co-culture of the MDM and neutrophils as described but without LPS. For neutrophils mono-culture, live or apoptotic neutrophils were plated in the same media, at the same density and in the same 96 well culture plate type (Falcon, 353072) as for the co-culture assay. The co- and mono- cultures were maintained for 6 h to mimic the standard co-culture assay time. After 6 h, the supernatants were removed and centrifuged at 300 g for 5 min to remove any cells or cell debris. For ultracentrifugation, the samples were spun at a further 100,000×*g* for 2 h (Beckman Optima TLX Ultracentrifuge). All supernatants were then added to the MDM and assayed for 6 h with or without LPS.

For phagocytosis, apoptotic neutrophils were labelled with CellTracker Green (Invitrogen) and pHrodo Red (Invitrogen). The labelled apoptotic neutrophils were then co-cultured with MDM at a ratio of 3:1 for 40 min at 37 °C with LPS (1 ng/ml). The co-cultures were then washed 3 times with HBSS prior to detachment of the cells with trypsin/EDTA and flow cytometric analysis (see below).

### Flow cytometry and cell sorting

Apoptosis was determined by flow cytometry (BD Biosciences LSR Fortessa) with cells stained for annexin IV-APC (BD Biosciences), propidium iodide (Sigma Aldrich) or active caspase 3/7 (CellEvent; Molecular Probes) and counter stained with Hoechst 3342 (NucBlue; Molecular Probes). Isolation of cell populations was performed using cell sorting (BD Biosciences FACs Aria II). BAL cells were stained with anti-HLA-DR V450, anti-CD-16 PE and CD3-FITC (BD Biosciences). AM were isolated by forward/side scatter gating on the HLA-DR positive population. Neutrophils were gated on CD16 positive cells which were HLA-DR and CD3 negative. Phagocytosis of apoptotic neutrophils was determined by flow cytometric analysis of MDM positive for CellTracker Green and pHrodo Red (BD Biosciences LSR Fortessa). Analysis of P-p65 ser529 in MDM by flow cytometry was performed using BD Biosciences PhosFlow reagents and protocol. See supplementary data for extended protocol.

### ELISA

Supernatants were removed from the cell cultures at the times indicated, centrifuged at 3800g. for 1 min to remove cells and subject to either immediate ELSA or stored at −20 °C until use. All ELISA were performed using DuoSet Kits (R&D Systems) according to manufacturer’s instructions. Plates were analysed on a Synergy HT Biotech plate reader using Gen 5 software.

### Immunobotting

Lysates were subject to gel electrophoresis using 5–12% Bis-Tris gels (Invitrogen), transferred to optitran nitrocellulaose membranes (Sigma) and blocked in TBS-0.1% tween with 5% non-fat dried milk. Primary antibodies were probed overnight in TBS-0.1% Tween containing 5% BSA with secondary antibodies probed in TBS-0.1% Tween containing 5% non-fat dried milk for 1 h. Immunoblots were developed using ECL prime (GE Life Sciences) on CL-XPosure film (Sigma) using an EcomaxProtec developer (Photon Imaging Systems). See supplementary materials and methods for extended protocol including cell lysis. Antibodies list: anti-P-p65 ser536 (#3031), anti-P-IKKα/β (#2697), anti-IKKβ (#8943), anti-P-TAK1 (#4508) and anti-TAK1 (#5206) were purchased from Cell Signalling Technology. Anti-p65 (Santa Cruz Biotechnology: sc-372), Anti-P-p65 ser529 (eBioscience: 14-9864-82), anti-P-p65 ser276 (Millipore: AB3375), anti-GAPDH (Sigma: G9295) and anti-β-Actin (Sigma: A1978). Goat anti-rabbit (P0448) and goat anti-mouse (P0447) secondary antibodies were purchased from Dako.

### ChIP assay

ChIP assays were performed using EZ Magna ChIP Kits (Millipore) according to the manufacturer’s instructions. Cells were fixed using 1% methanol free formaldehyde (Pierce; ThermoFischer Scientific) and subject to sonication for 10 cycles of 15 s (Soniprep 150; MSE). IP was performed using the ChIPAb+NF-κB p65 antibody (Millipore) and qPCR performed using ChIP-qPCR Primer Assay for Human TNF (SABiosciences) with SYBR Green qPCR master mix (SABiosciences) and ran on a 7900HT Fast RT-PCR System (Applied Biosystems) with analysis performed using Applied Biosystems SDS 2.4 software.

### Immunocytochemistry

Staining for p65 was performed using a modified protocol from Blaecke A. et al^37^ using Lab-Tek chamber slides (Nunc; Sigma) for confocal microscopy, or 96 well optical image plates (Greiner) for high content imaging. Cells were fixed with 4% PFA (Sigma), permeabilised with methanol then stained with anti-p65 antibody (sc-372-G; Santa Cruz Biotechnology), Donkey anti-Goat IgG AF647 (Molecular probes A-21447), Hoechst 33342 (NucBlue; Molecular Probes) and phalloidin (Molecular Probes). Confocal microscopy was performed using a Zeiss LSM510 META and high content imaging performed using an ImageXpress XLS Widefield High-Content Analysis System (Molecular Devices) with MetaXpress analysis. See supplementary data for extended protocol.

### Gene array

Cells were washed with ice cold PBS, incubated in scrape buffer (see immunoblotting) for 10 min before being scraped then pelleted at 300×*g* for 5 min at 4 °C. RNA extraction was performed using RNeasy Mini Kits (Qiagen) according to the manufacturer’s instructions. An Affimetrix human genome U219 array was performed by Edinburgh Genomics (https://genomics.ed.ac.uk/) with Partek Software analysis. The data discussed in this publication have been deposited in NCBI’s Gene Expression Omnibus (GEO) and are accessible through GEO Series accession number GSE90010 (http://www.ncbi.nlm.nih.gov/geo/query/acc.cgi?acc=GSE90010).

### Data analysis

Comparison of multiple treatment groups was performed using one-way ANOVA with Tukeys post-hoc test, comparisons between two treatment groups was performed using paired student’s *t*-test and the data presented as the mean ± s.e.m. Analysis was performed using GraphPad Prism Software.

## Electronic supplementary material

Supplementary data figure 1

Supplementary data figure 2

Supplementary data figure 3

Supplementary data figure 4

Supplementary data table 1

Supplementary extended materials and methods
