# Supplementary figures and images for: Neutrophils induce macrophage anti-inflammatory reprogramming by suppressing NF-κB activation
Source: Cell Death Dis. 2018 Jun 4;9(6):665. doi: 10.1038/s41419-018-0710-y (PMC5986789; doi:10.1038/s41419-018-0710-y)

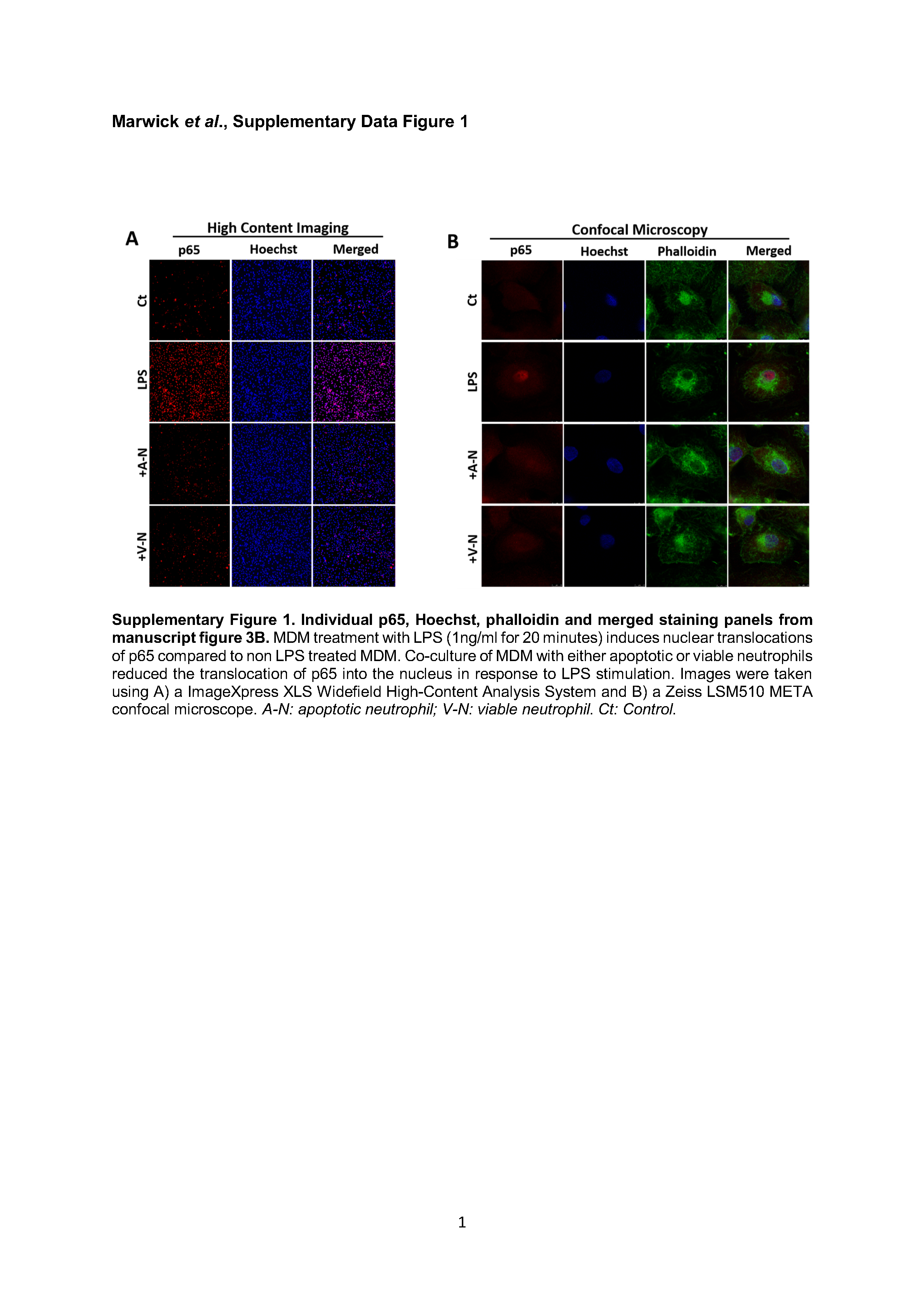

Supplement: Supplementary file 1 — Supplementary data figure 1 [file 41419_2018_710_MOESM1_ESM.tif]

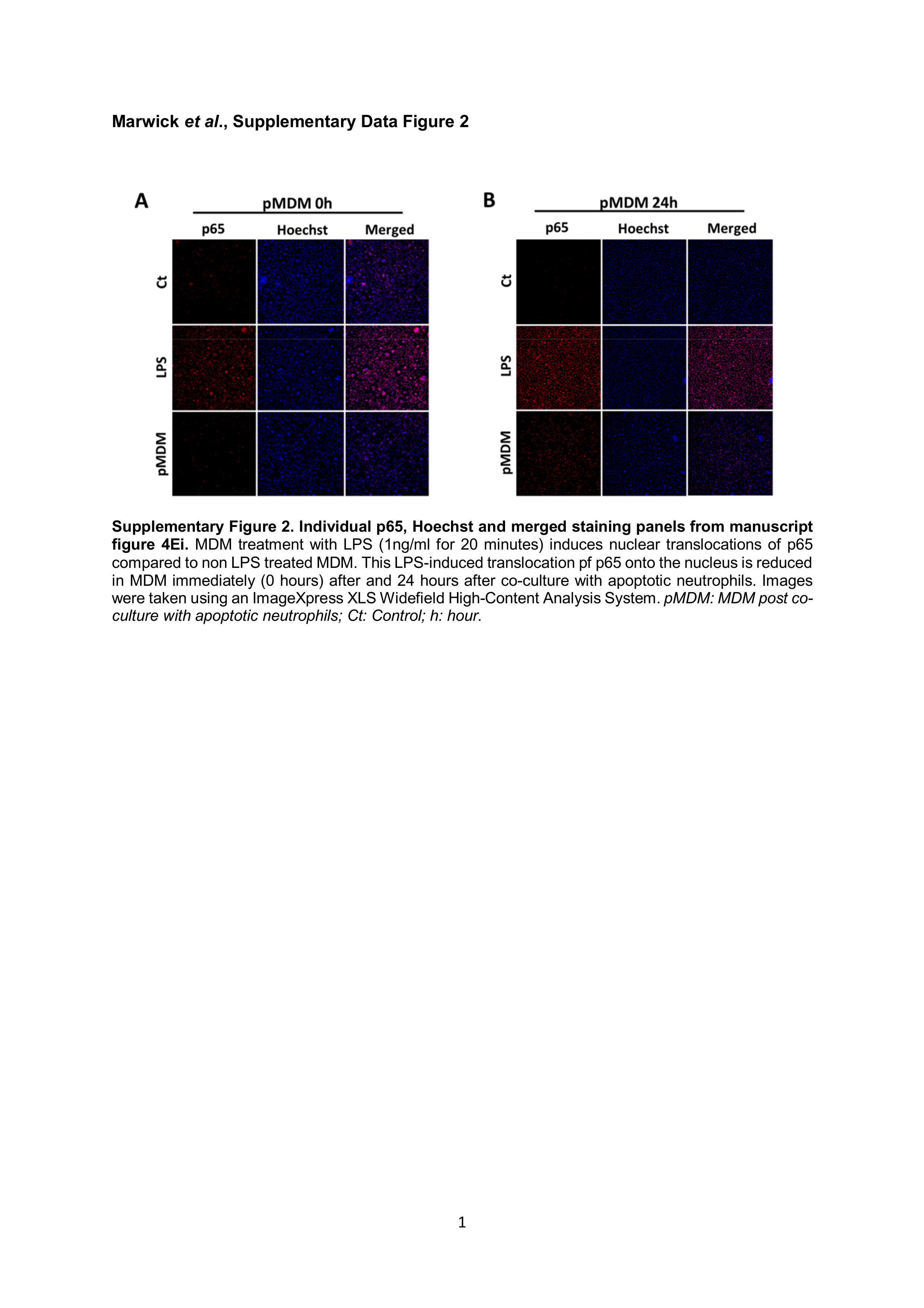

Supplement: Supplementary file 2 — Supplementary data figure 2 [file 41419_2018_710_MOESM2_ESM.tif]

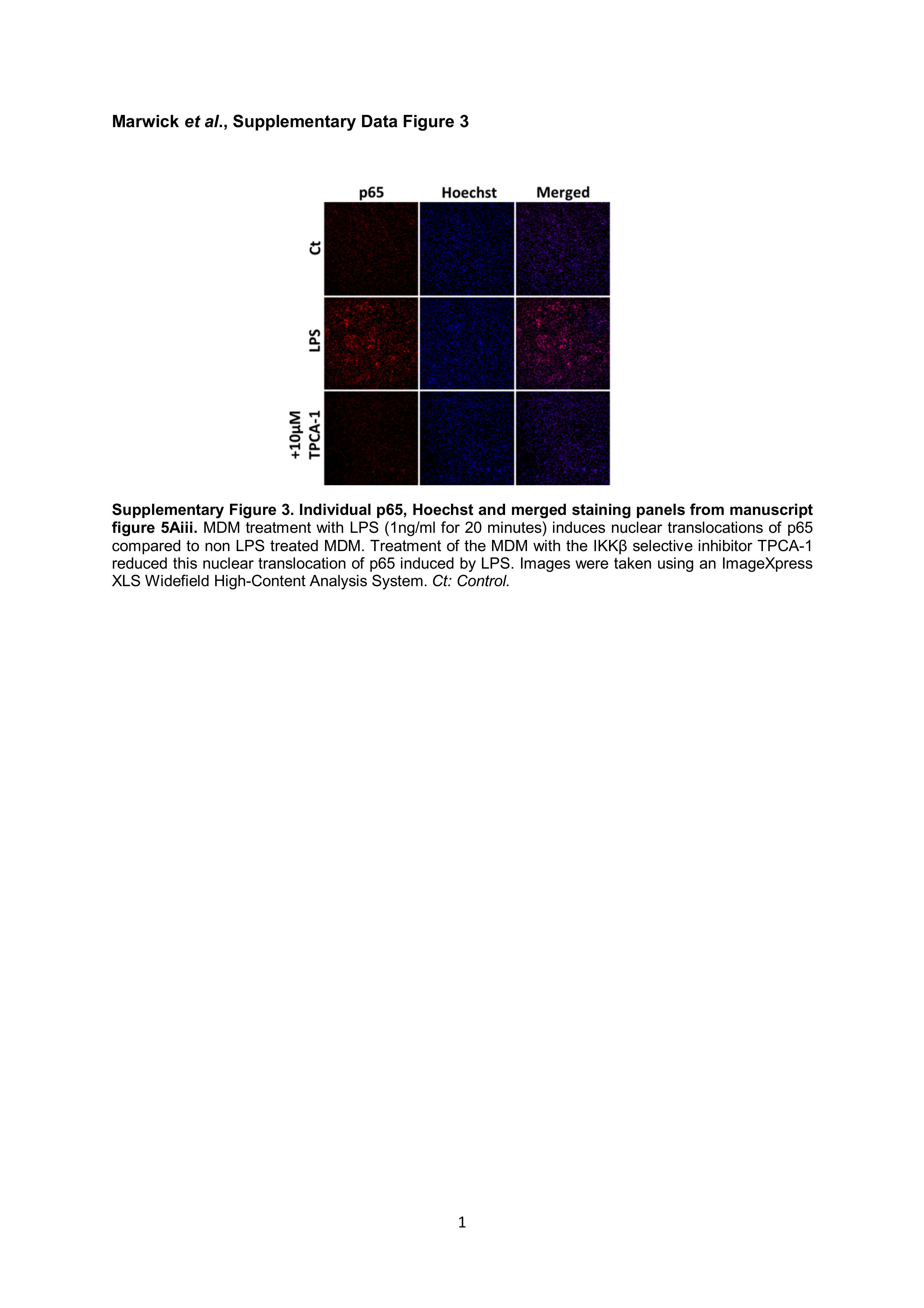

Supplement: Supplementary file 3 — Supplementary data figure 3 [file 41419_2018_710_MOESM3_ESM.tif]

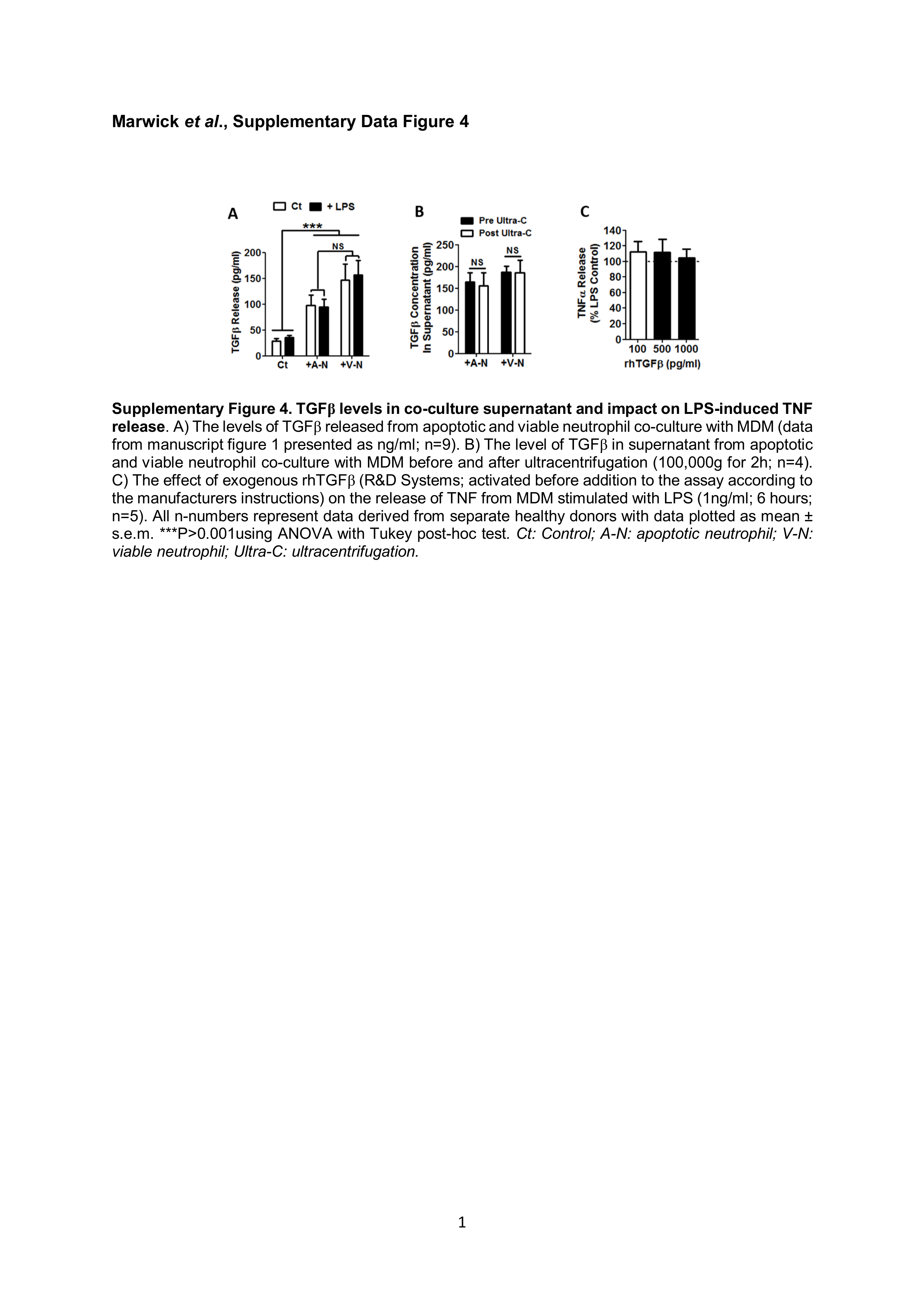

Supplement: Supplementary file 4 — Supplementary data figure 4 [file 41419_2018_710_MOESM4_ESM.tif]

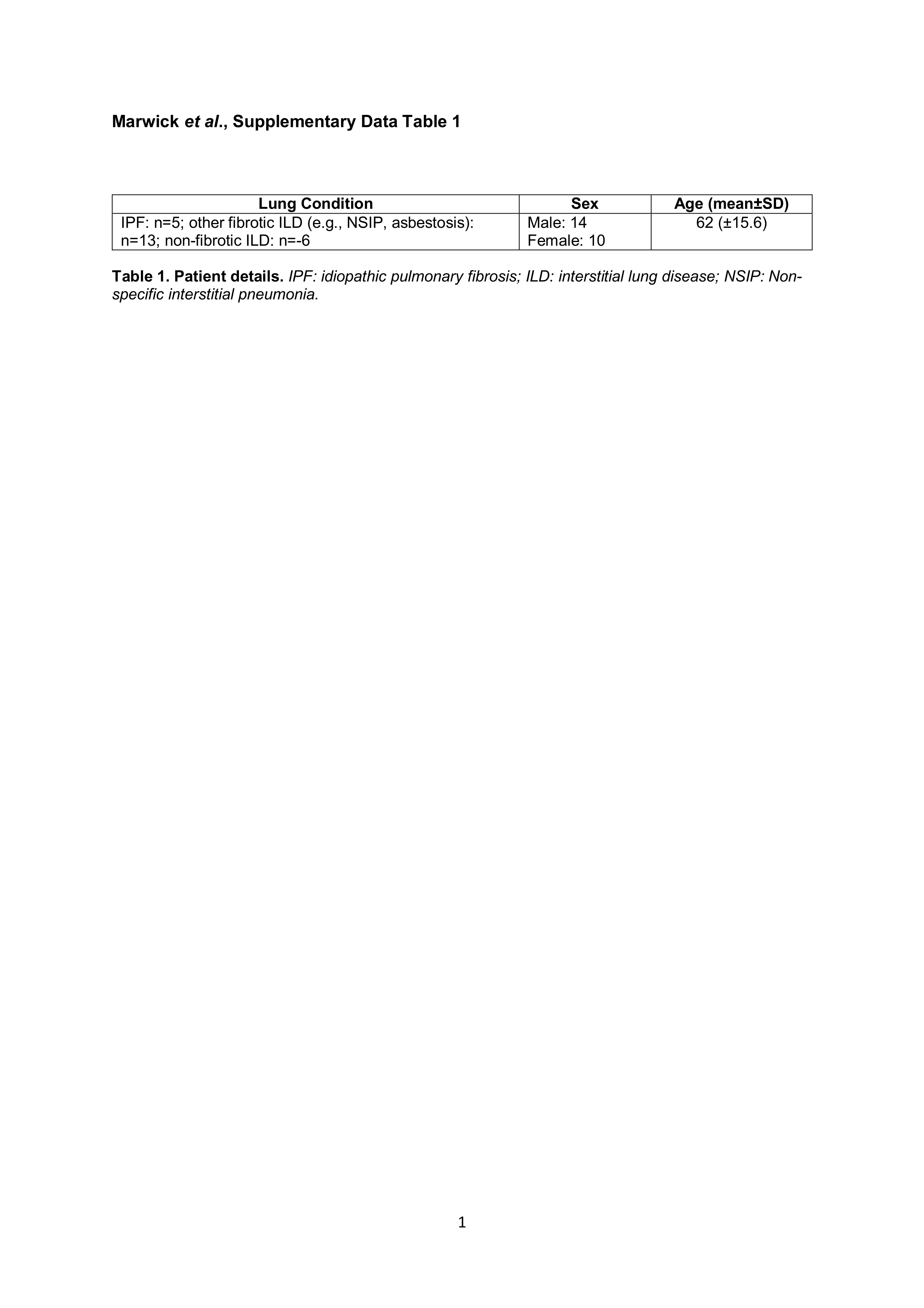

Supplement: Supplementary file 5 — Supplementary data table 1 [file 41419_2018_710_MOESM5_ESM.tif]
