# Supplementary material for: Neutrophils induce macrophage anti-inflammatory reprogramming by suppressing NF-κB activation
Source: Cell Death Dis. 2018 Jun 4;9(6):665. doi: 10.1038/s41419-018-0710-y (PMC5986789; doi:10.1038/s41419-018-0710-y)
Supplement: Supplementary file 6 — Supplementary extended materials and methods [file 41419_2018_710_MOESM6_ESM.pdf]

Marwick *et al*., Supplementary Data

Extended Materials and Methods

Flow cytometry & cell sorting: Apoptosis was determined in neutrophils, BL-2 and jurkat cells using flow cytometry (BD Biosciences LSR Fortessa) by labelling the cells with annexin IV-APC (BD Biosciences) and propidium iodide (Sigma Aldrich) or active caspase 3/7 (CellEvent; Molecular Probes) and counter stained with Hoechst 3342 (NucBlue; Molecular Probes). BAL cells were stained with anti-HLA-DR V450, anti-CD-16 PE and anti-CD3-FITC (BD Biosciences) for cell sorting (BD Biosciences FACs Aria II). AM were isolated by gating on the HLA-DR positive population then using forward/side scatter. Neutrophils were HLA-DR and CD3 negative, but strongly positive for CD16. Phosphorylation of p65 on serine 529 (Pp65 ser529) was determined by flow cytometry (BD Biosciences LSR Fortessa) using BD PhosFlow reagents and protocol. Briefly, MDM differentiated and co-cultured with A-N or V-N on square 25 well petri culture dishes (Sterilin; Fischer Scientific) for 10 minutes, followed by stimulation with LPS for 20 minutes. The co-cultures were then placed on ice, washed with ice-cold PBS, the incubated in ice-cold PBS containing Phosphatase Cocktail Inhibitor II (Merck-Millipore) for 10 minutes on ice. The MDM were then fixed using Cytofix Buffer (BD Biosciences), washed using Stain Buffer (BD Biosciences) then scraped and pelleted, permeablised using Perm Buffer III (BD Biosciences), incubated with anti-P-p65 or IgG Control (BD Biosciences) for 25 minutes at RT then analysed

Immunobotting: Cells were placed on ice for 5 minutes, washed with ice-cold PBS then incubated for a further 10 minutes in scrape buffer (PBS with 0.1% BSA, 2mM EDTA, Phosphatase Cocktail Inhibitor II and Complete Protease Inhibitor Mini (Sigma) before being scrapped, pelleted (300g for 5 minutes at 4˚C). For cytosolic and nuclear/extraction, pellets were re-suspended in a hypotonic cytosolic extraction buffer (50mM Tris-HCl pH8.0, 10mM $MgCl_2$, 1mM EDTA, 0.05% NP-40, 10% glycerol, 0.5mM AESBF, 10ug/ml aprotinin, 10ug/ml leupeptin, 10ug/ml pepstatin A, 1mM sodium orthovanadate, 0.5mM benzamidine, 2mM levamisole, 10mM β-glycerophosphate and protease inhibitor cocktail (P8340; Sigma) for 15 minutes on ice before being centrifuged at 13,000rpm for 20 minutes. The nuclear pellet was washed 3x in cytosolic extraction buffer before being lysed using a modified RIPA buffer (50mM Tris-HCL pH 7.6, 150mM NaCl, 1mM EDTA, 0.5% SDS, 0.5% deoxycholic acid, 1% NP-40, 0.5mM AEBSF,10ug/ml aprotinin, 10ug/ml leupeptin, 10ug/ml pepstatin A, 1mM sodium orthovanadate, 0.5mM benzamidine, 2mM levamisole, 10mM β-glycerophosphate and protease inhibitor cocktail (P8340; Sigma). For total cell lysis, pellets were lysed using the modified RIPA buffer. Lysates were denatured by boiling for 3 minutes in Novex SDS loading buffer (Invitrogen) containing β-mercaptoethanol (Sigma). Denatured lysates were subject to gel electrophoresis using 5-12% Bis-Tris gels (Invitrogen) and transferred to Optitran nitrocellulose membranes (Sigma). Membranes were blocked in Tris-buffered saline (TBS) containing 5% non-fat dried milk and 0.1% Tween 20 (Sigma). Blocked membranes were then washed in TBS-0.1% Tween for 5 minutes before being probed over night with the primary antibody in TBS-0.1% Tween containing 5% BSA. Membranes were washed (3 x 10 minutes TBS-0.1% Tween), probed with secondary antibody (Dako), washed then developed with ECL prime (GE Life Sciences) and CL-XPosure film (Sigma) using an EcomaxProtec developer (Photon Imaging Systems). Antibodies list: anti-P-p65 ser536 (#3031), anti-P-IKKα/β (#2697), anti-IKKβ (#8943), anti-P-TAK1 (#4508) and anti-TAK1 (#5206) were purchased from Cell Signalling Technology. Anti-p65 (Santa Cruz Biotechnology: sc-372), Anti-P-p65 ser529 (eBioscience: 14-9864-82), anti-P-p65 ser276 (Millipore: AB3375), anti-GAPDH (Sigma: G9295) and anti-β-Actin (Sigma: A1978). HRP-conjugated goat anti-rabbit IgG (P0448) and goat anti-mouse IgG (P0447) secondary antibodies were purchased from Dako.

Immunocytochemistry: MDM were co-cultured with apoptotic or viable neutrophils for 20 minutes in Lab-Tek chamber slides (Nunc; Sigma) for confocal microscopy, or 96 well optical image plates (Greiner) for high content imaging. The co-cultures were stimulated with LPS (1ng/ml) for 40 minutes, washed 3 times with PBS (Gibco), fixed with 4% PFA (Sigma) for 15 minutes at room temperature (RT), washed 3 times with PBS and then permeabalised using ice-cold methanol for 5 minutes at -20°C. The MDM were then washed 3 times with Stain Buffer (BD Biosciences), blocked for 10 minutes with Stain Buffer followed by incubation with anti-p65 antibody (sc-372-G; Santa Cruz Biotechnology) for at 10µg/ml in Stain Buffer for 1 hour at RT. The MDM were then washed 3 times with Stain Buffer and incubated with Donkey anti-goat IgG (H+L) AF647 (Molecular probes A-21447) at 10µg/ml in Stain Buffer for 1 hour at RT. Hoechst 33342 (NucBlue; Molecular Probes) was added for last 10 minutes of

the incubation. For confocal microscopy, the MDM were also stained with phalloidin (Molecular Probes) for 30min in Stain Buffer. Following staining, the MDM were then washed 3 times in PBS. For confocal microscopy, the chamber slides were mounted using ProLong Gold Antifade (Molecular Probes) mounting media then imaged using a Zeiss LSM510 META. For High content imaging, the cells were kept in PBS and imaged using an ImageXpress XLS Widefield High-Content Analysis System (Molecular Devices) with MetaXpress analysis.
